# Time-series transcriptomic analysis of flower tissue during heat stress in heat-resilient and heat-sensitive *Brassica napus* L.

**DOI:** 10.3389/fgene.2025.1736538

**Published:** 2026-01-12

**Authors:** Xiaojie Hu, Sheng Chen, Xiaoke Ping, Kadambot H. M. Siddique, Wallace A. Cowling

**Affiliations:** 1 The UWA Institute of Agriculture, The University of Western Australia, Perth, WA, Australia; 2 School of Agriculture and Environment, The University of Western Australia, Perth, WA, Australia; 3 College of Agronomy and Biotechnology, Southwest University, Chongqing, China

**Keywords:** *Brassica napus*, canola, differentially expressed genes, heat resilience, heat stress, oilseed rape, post-pollination, transcriptomics

## Abstract

*Brassica napus* a cool-season oilseed crop, is an important source of edible oil and biofuel. Heat stress during the reproductive stage poses a serious threat to its productivity, but little is known about the gene networks involved in the heat stress response during this phase. In this study, we conducted a time-series transcriptome analysis of heat-stress response in flowers and immature pods of four cultivars of *B. napus* to reveal heat-responsive gene pathways associated with heat sensitivity and resilience. Individual plants were moved to the heat stress or control treatments in the morning of the day when the flower opened at the second reproductive node of the main stem, defined as day zero after treatment (DAT0). Flowers at the second to fifth reproductive nodes on the main stem were collected in the afternoons of DAT0, DAT1, DAT3 and DAT6 of heat stress and control treatments for time-series transcriptome analysis. A total of 36,933 differentially expressed genes (DEGs) were identified in reproductive tissue under heat stress compared to the control treatment. In heat-resilient cultivar AV-Ruby, more than 4,000 unique DEGs were enriched in DNA repair and antioxidant defence pathways which support efficient stress recovery mechanisms and enhanced protection against oxidative damage in flower and immature pods tissue. Three heat shock proteins were upregulated under heat stress in all four cultivars of *B. napus* from fertilisation to early embryo and seed development, which highlights their core role in the heat stress response. The unique temporal responses to heat stress in heat-resilient and heat-sensitive cultivars provides a foundation for understanding heat stress resilience during the reproductive stage.

## 1 Introduction

Heat stress during the reproductive stage significantly impairs seed formation and thus threatens crop yield (Zenda et al., 2022; Qian et al., 2025). In *Brassica napus* L., exposure to high temperatures during this critical phase can result in substantial yield losses (Chen et al., 2021; Pokharel et al., 2021; Hu et al., 2024; Hu et al., 2025; Mohammadi et al., 2025). Temperatures above 29.5 °C during flowering have been shown to drastically reduce yield (Morrison and Stewart, 2002). As heatwaves become more frequent in major growing regions such as Canada and Australia, heat stress has become a major constraint on *B. napus* productivity (Sadras and Dreccer, 2015; Breshears et al., 2021; Koscielny and Duncan, 2024).

Although some *B. napus* cultivars show greater resilience to heat with minimal yield reductions (Mácová et al., 2022; Mohammadi et al., 2025), the underlying mechanisms driving this variation remain unclear. Plant acclimation to stress involves perception and transmission of signals, followed by physiological and biochemical adjustments (Firmansyah and Argosubekti, 2020; Shekhawat et al., 2022). Transcriptomic responses often represent early responses that regulate downstream protein expression and metabolism to maintain cellular homeostasis.

Transcriptomic studies provide evidence of the cellular and molecular responses underlying plant adaptation to environmental stresses (Zhang et al., 2021). RNA sequencing (RNA-seq) is a powerful transcriptomic technique that uses next-generation sequencing to reveal the presence and quantity of RNA molecules in biological samples. It has been widely applied to explore molecular responses to heat stress in various plant species, including garlic (Yang et al., 2024), cotton (Masoomi-Aladizgeh et al., 2022) and pepper (Wang et al., 2021). While transcriptomic analyses have been conducted in *B. napus* exposed to heat stress, these focused on limited time points, without exploring the time-dependent changes of gene expression (Yu et al., 2014; Huang et al., 2019).

Among other reactions, heat stress stimulates production of functional proteins known as heat shock proteins (HSPs), and many of HSP-related gene sequences have been found in Brassicaceae (Cantila et al., 2024). Among other differentially abundant proteins (DAPs) in *B. napus*, several HSPs were reported by Hu et al. (2025) to be abundant across all cultivars and time-points during heat stress. However, there is a gap in knowledge of differentially expressed genes (DEGs) and molecular mechanisms in *B. napus* in response to heat stress, especially during the heat-sensitive stage of fertilisation and immature pod development.

To address this gap, we evaluated DEGs during 7 days of heat or control temperature treatment after pollination of flowers at the second to fifth reproductive nodes on the main stem. This time series transcriptomics experiment ensured that flower tissue was exposed to exactly the same temperature regimes at the same flower age and position on the plant. We hypothesised that differential gene expression heat stress is mediated by dynamic regulatory networks that change over these seven critical days of flower and seed development, and differ between heat resilient and sensitive cultivars. This study will improve our knowledge of common and cultivar-specific heat response pathways associated with heat resilience and sensitivity at this critical stage of reproduction in *B. napus*, and guide the future breeding of heat-tolerant *B. napus*.

## 2 Materials and methods

### 2.1 Plant material and heat treatments

This experiment was conducted in a screenhouse equipped with two heat-treatment chambers at The University of Western Australia’s Shenton Park Field Station, Perth, Western Australia (31°56′54″ S, 115°47′44″ E). Four *B. napus* cultivars were used, including two early-flower cultivars (AV-Ruby and ZY821), and two late-flower cultivars (Alku and YM11) (Chen et al., 2023). To synchronize anthesis across all cultivars, the early-flowering cultivars were sown 4 weeks later than the late-flowering ones.

Plants were grown in the screenhouse until the second flower on the main stem was open. At 08:00 on that day, the 2nd to 5th flowers on the main stem were pollinated with pollen collected from freshly opened flowers of the same cultivar. Then the plants were covered with selfing bags and moved to two separate heat treatment chambers for 7 days, with a control treatment with transient daily maximum/minimum temperatures of 25 °C/15 °C, and a heat stress treatment with transient maximum/minimum temperatures of 32 °C/22 °C. The plant management and chamber settings followed the protocols described in Hu et al. (2025).

After temperature treatment, plants were returned to the screenhouse and grown to maturity. At maturity, pods formed from the 2nd to 5th flowers on the main stem were collected and dried at 30 °C for 14 days. Yield-related traits, including pod length, seed number per pod, seed yield per pod and 1,000-seed weight were measured in these pods.

For transcriptome analysis, the 2nd to 5th flowers and immature pods on the main stem were pooled into a single sample and collected at 14:00 on day zero after beginning of heat treatment (DAT0), DAT1, DAT3, and DAT6, following the protocol described in Hu et al. (2025). Samples were immediately frozen in liquid nitrogen and stored at −80 °C. This experiment included three biological replicates of four cultivars sampled at four time points under both heat and control conditions, resulting in a total of 96 samples.

### 2.2 Total RNA extraction and RNA sequencing

Total RNA extraction was followed the same methods described in Hu et al. (2025) from each of the 96 samples. A total of 10 μg of RNA from each sample was aliquoted and sent to Biomarker Technologies (BMKGENE, Hongkong, China) for mRNA purification, cDNA library construction and sequencing by using Illumina technology.

RNA sequencing was performed using the Illumina Novaseq X platform (Illumina, San Diego, CA, United States), generating high-quality sequencing reads. Raw sequencing data in FASTQ format were initially processed using in-house Perl scripts to filter out adapter sequences, ploy-N-containing reads, and other low-quality reads. After this quality control step, clean reads were obtained, and their quality metrics, Q20, Q30, GC-content and sequence duplication levels were calculated.

The clean reads were then mapped to the *B. napus* reference genome (v4.1) (https://www.genoscope.cns.fr/brassicanapus/data/Brassica_napus_v4.1.chromosomes.fa.gz). Only reads with no more than one single mismatch were retained for further analysis and gene annotation.

### 2.3 Gene expression quantification and DEGs identification

The expression levels of genes were quantified using fragments per kilobase of transcript per million mapped reads (FPKM). Gene expression levels were compared between heat and control treatments. Differential expression analysis was conducted by DESeq2 (Love et al., 2014). The *p*-values were adjusted using the Benjamini and Hochberg’s approach to control the false discovery rate (FDR) (Benjamini and Hochberg, 1995). DEGs were considered significant if they met the threshold of fold change ≥1.5 and FDR <0.01. Venn diagrams were generated by using the Molbiotools (https://molbiotools.com).

### 2.4 Bioinformatics analysis

Gene function annotation was conducted with multiple databases, including COG (Clusters of Orthologous Genes), GO (Gene Ontology), KEGG (Kyoto Encyclopedia of Genes and Genomes), KOG (Eukaryotic Orthologous Groups of Proteins), Pfam (Protein family), SWISS-PROT (a curated protein sequence database), eggNOG database (evolutionary genealogy of genes: Non-supervised Orthologous Groups), and NCBI-nr (NCBI non-redundant protein sequences).

Functional enrichment analysis of GO terms and KEGG pathways were performed at the *Brassica napus* multi-omics information resource (BnIR) (https://yanglab.hzau.edu.cn/BnIR) (Yang et al., 2023). Short time-series expression miner (STEM) analysis was performed using the OmicShare tools (www.omicshare.com/tools) (Mu et al., 2024). The STEM clustering method was applied to analyse the expression patterns of AV-Ruby specific DEGs, based on the FPKM at four time points under heat treatment. The number of model profiles was set to 10, and the threshold of significant profile was set as *p* < 0.05.

### 2.5 Quantitative real-time PCR validation

Quantitative real-time PCR (qRT-PCR) was performed to validate the RNA-Seq findings. One ug RNA of each sample was reverse-transcribed using iScript™ gDNA Clear cDNA Synthesis Kit (Bio-Rad, Hercules, CA, United States). Thirteen DEGs were randomly selected across four time points for the qRT-PCR experiment: *BnaA10g20610D*, which showed the highest average expression among the three consistently upregulated DEGs, *BnaA01g23000D*, *BnaA02g28330D*, *BnaA02g36510D*, *BnaA05g09400D*, *BnaA09g52180D*, *BnaC04g13110D*, *BnaC04g22890D*, *BnaC07g39860D*, *BnaC09g16520D*, *BnaC09g45670D*, *BnaCnng12730D*, and *BnaCnng52120D*. Primers were designed for each DEG using the NCBI online website Primer-BLAST (http://www.ncbi.nlm.nih.gov/tools/primerblast) (Supplementary Table S1). *BnActin7* served as an internal control for normalizing gene expression levels (Chen et al., 2010). qRT-PCR was performed with iTaq™ Universal SYBR^®^ Green Supermix (BioRad, Hercules, CA, United States). All experiments were carried out at least three times on three biological replicates. The relative expression levels were calculated by the method of fold change of 2^−ΔΔCT^ value (Schmittgen and Livak, 2008).

### 2.6 Statistical analysis

Analysis of variance (ANOVA) was performed using R software (version 4.3.3) with the ‘tidyverse’ R package (version 2.0.0) (Wickham et al., 2019; R Core Team, 2024). Two-way ANOVA was employed to assess interactions, and pairwise comparisons of means were conducted using the least significant difference (LSD) test. A difference between two means greater than the LSD value was considered significantly different at *p* < 0.05 (Butler, 2022).

## 3 Results

### 3.1 Effect of heat treatment during post-pollination stage

All hand-pollinated flowers formed fertile pods at maturity, and each pod contained at least one seed. Highly significant main effects of cultivar (C) were observed for all four yield traits (*p* < 0.001), while significant main effects of heat treatment (T) were detected for pod length (*p* < 0.001), number of seeds per pod (*p* < 0.001) and seed yield per pod (*p* < 0.001), but not for 1,000-seed weight. Additionally, significant main effects of C × T interaction (*p* < 0.05) were observed in pod length and seed yield per pod (Supplementary Table S2).

**FIGURE 1:**
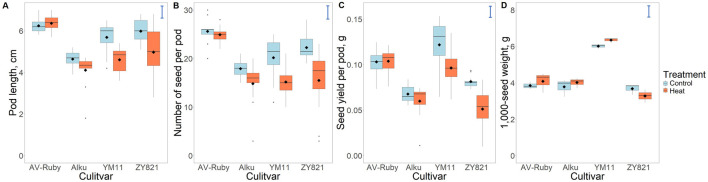
Box plots showing the response to heat stress of yield-related traits in the 2nd to 5th reproductive nodes on the main stem of *Brassica napus* cultivars: AV-Ruby, Alku, YM11 and ZY821, including **(A)** pod length (cm), **(B)** number of seeds per pod, **(C)** seed yield per pod (g), and **(D)** 1,000-seed weight (g). For each cultivar, the response is compared after 7 days of control conditions (daily maximum/minimum temperatures of 25 °C/15 °C) and heat stress conditions (daily maximum/minimum temperatures of 32 °C/22 °C) during flowering in the 2nd to 5th reproductive nodes on the main stem. Lower and upper box boundaries indicate the 25th and 75th percentiles. Lines and black diamonds within boxes represent medians and means, respectively. Whiskers extend to 1.5 times the interquartile range, and black dots indicate outliers. Error bars represent least significant difference values between any pair of means at the 5% significance level (for ANOVA, see Supplementary Table S2).

Among the four cultivars, AV-Ruby, showed no significant reduction in any yield-related traits under heat compared to control treatment in flowers 2–5 (Figure 1; Supplementary Table S3). In contrast, YM11 and ZY821 showed a significant reduction in pod length after heat treatment, at 81.1% and 83.1% of the control, respectively. Moreover, Alku, YM11 and ZY821 experienced a notable decline in the number of seeds per pod and seed yield per pod after heat stress. ZY821 was the most heat-sensitive cultivar with seed yield per pod in the heat treatment was only 62.8% of control. However, 1,000-seed weight was not significantly affected by heat treatment in all four cultivars (Figure 1; Supplementary Table S3).

### 3.2 Overview of sequencing data analysis

After filtering and trimming, an average of 46.7 million high-quality clean reads were retained for further analysis. The average Q20 and Q30 values were 97.7% and 93.6%, respectively (Supplementary Table S4). The clean reads were mapped to the *B. napus* reference genome with an average mapping rate of 87.78%.

### 3.3 Gene function annotation

Functional annotation was performed using multiple databases, with the following number of genes assigned: 26,566 (22.7%) to COG, 74,968 (63.9%) to GO, 63,879 (54.5%) to KEGG, 50,575 (43.1%) to KOG, 73,628 (62.8%) to Pfam, 69,008 (58.9%) to SWISS-PROT, 77,641 (66.2%) to eggNOG, and 109,161 (93.1%) to NCBI-nr. In total, 109,317 (93.2%) genes were successfully annotated across these databases (Supplementary Table S5).

### 3.4 Identification of differentially expressed genes (DEGs)

**FIGURE 2:**
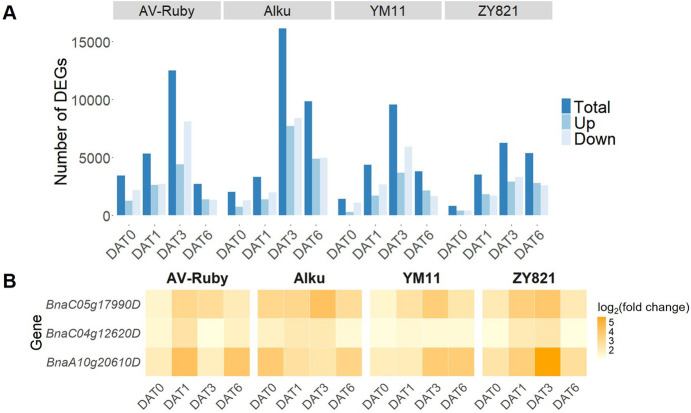
**(A)** The number of differentially expressed genes (DEGs) identified in four *Brassica napus* cultivars: AV-Ruby, Alku, YM11, and ZY821, between control (daily maximum/minimum temperatures of 25 °C/15 °C) and heat stress (daily maximum/minimum temperatures of 32 °C/22 °C) treatments at four time points: DAT0 (day zero of heat treatment), DAT1, DAT3, and DAT6. **(B)** Gene expression level (log_2_[fold change]) for three common heat shock proteins that were consistently upregulated across all cultivars and time points. For gene functions see Supplementary Table S6.

Across the 4 sampling times and 4 cultivars, a total of 36,933 DEGs were identified in heat vs*.* control treatments (Supplementary Table S6). The total number of DEGs followed a regular temporal pattern across the four cultivars, with an increase from DAT0 to DAT3, then a decline from DAT3 to DAT6. This pattern occurred in both upregulated and downregulated DEGs which reached peak levels at DAT3 in all four cultivars. At DAT0, AV-Ruby exhibited the highest number of total DEGs, including both up- and downregulated DEGs, among the four cultivars. At DAT0, a total of 3,436 DEGs were identified in AV-Ruby, with 1,270 upregulated and 2,166 downregulated. On the contrary, at DAT6, AV-Ruby displayed the lowest DEG count (2,713 in total) among the four cultivars (Figure 2A).

Three DEGs - *BnaC05g17990D*, *BnaC04g12620D*, and *BnaA10g20610D* - encoded HSP and HSP-like proteins and were consistently upregulated across all four time points and in all four cultivars (Supplementary Table S6).

*BnaC05g17990D* encoded a HSP20-like chaperones superfamily protein. In AV-Ruby, *BnaC05g17990D* peaked earlier than in the other three cultivars at DAT1 (log_2_[fold change] = 3.06), while its peak expression occurred at DAT3 in Alku (log_2_[fold change] = 4.18), YM11 (log_2_[fold change] = 3.55), and ZY821 (log_2_[fold change] = 3.84) (Figure 2B; Supplementary Table S6).

*BnaC04g12620D* encoded a 70-kDa HSPA8, and was consistently upregulated in YM11 across all time points (average log_2_[fold change] = 1.30) (Figure 2B; Supplementary Table S6).

*BnaA10g20610D* encoded a 17.6-kDa class II HSP, and exhibited the highest average expression levels among these three genes, but its expression varied considerably across all cultivars and time points. YM11 showed an increasing trend over time, while the highest expression was detected at DAT0 in Alku (log_2_[fold change] = 3.74), at DAT1 in AV-Ruby (log_2_[fold change] = 4.21), and at DAT3 in ZY821 (log_2_[fold change] = 5.67) (Figure 2B; Supplementary Table S6).

### 3.5 GO terms and KEGG pathway enrichment across four time points

**FIGURE 3:**
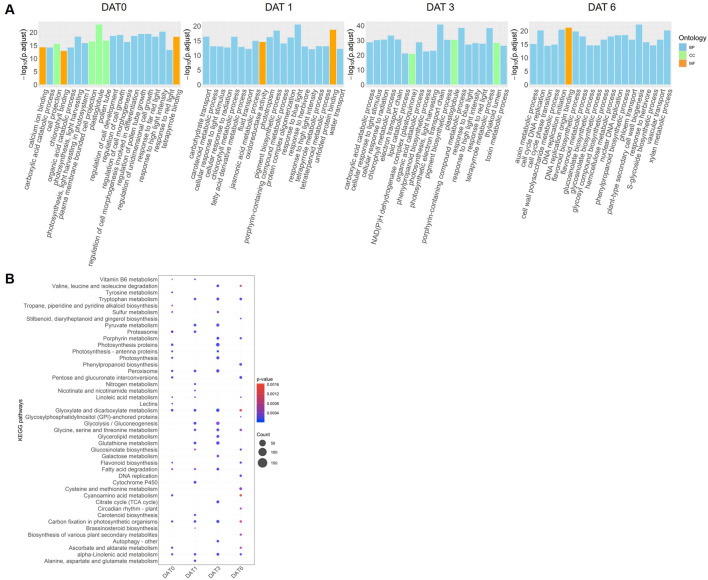
The functional enrichment analysis of the differentially expressed genes (DEGs) across four *Brassica napus* cultivars (AV-Ruby, Alku, YM11, and ZY821) obtained at four time points: day zero of heat treatment (DAT0), DAT1, DAT3, and DAT6. **(A)** The top 20 significant enriched Gene Ontology (GO) terms (ranked by -log10[adjusted *p*-value] of expression value). The GO terms were assigned to three categories: biological process (BP), cellular component (CC), and molecular function (MF). **(B)** The top 20 significant enriched Kyoto Encyclopedia of Genes and Genomes (KEGG) pathways (ranked by *p*-value). The size of the dot shows the number of genes in each KEGG pathway. The dot colour represents the *p*-value (red is higher, blue is lower).

At DAT0, DEGs were significantly enriched in several GO terms related to cell growth and development and to pollen tube growth. At DAT1, DEGs were notably enriched in GO terms related to unfolded protein binding and hormonal signalling. At DAT3, DEGs were enriched in biological process. Notably, response to high light intensity DEGs were consistently enriched across DAT0, DAT1, and DAT3 (Table 1; Figure 3A).

**TABLE 1:** Gene ontology (GO) terms enriched on day 0, 1, 3 and 6 after heat stress and control treatments (DAT0, DAT1, DAT3 and DAT6) in four cultivars of *Brassica napus*.

DAT	GO term	Related to	Annotation
DAT0	GO:0051510	Cell growth and development	Regulation of unidimensional cell growth
DAT0	GO:0001558	Cell growth and development	Regulation of cell growth
DAT0	GO:0010769	Cell growth and development	Regulation of cell morphogenesis involved in differentiation
DAT0	GO:0060284	Cell growth and development	Regulation of cell development
DAT0	GO:0022604	Cell growth and development	Regulation of cell morphogenesis
DAT0	GO:0080092	Pollen tube growth	Regulation of pollen tube growth
DAT0	GO:0090406	Pollen tube growth	Pollen tube
DAT1	GO:0051082	Unfolded protein binding and hormonal signalling	Unfolded protein binding
DAT1	GO:0009694	Unfolded protein binding and hormonal signalling	Jasmonic acid metabolic process
DAT3	GO:0009767	Biological process	Photosynthetic electron transport chain
DAT3	GO:0006778	Biological process	Porphyrin-containing compound metabolic process
DAT3	GO:0033013	Biological process	Tetrapyrrole metabolic process
DAT0	GO:0010218	Response to high light intensity	Response to far red light
DAT1	GO:0071482	Response to high light intensity	Cellular response to light stimulus
DAT1	GO:0009644	Response to high light intensity	Response to high light intensity
DAT1	GO:0009637	Response to high light intensity	Response to blue light
DAT3	GO:0071482	Response to high light intensity	Cellular response to light stimulus
DAT3	GO:0009644	Response to high light intensity	Response to high light intensity
DAT3	GO:0009637	Response to high light intensity	Response to blue light
DAT6	GO:0045491	Secondary metabolites	Xylan metabolic process
DAT6	GO:0009813	Secondary metabolites	Flavonoid biosynthetic process
DAT6	GO:0009699	Secondary metabolites	Phenylpropanoid biosynthetic process
DAT6	GO:0009812	Secondary metabolites	Flavonoid metabolic process
DAT6	GO:0010410	Secondary metabolites	Hemicellulose metabolic process
DAT6	GO:1901659	Secondary metabolites	Glycosyl compound biosynthetic process
DAT6	GO:0009850	Secondary metabolites	Auxin metabolic process
DAT6	GO:0010383	Secondary metabolites	Cell wall polysaccharide metabolic process
DAT6	GO:0016144	Secondary metabolites	S-glycoside biosynthetic process
DAT6	GO:0019758	Secondary metabolites	Glycosinolate biosynthetic process
DAT6	GO:0019761	Secondary metabolites	Glucosinolate biosynthetic process
DAT6	GO:0003688	DNA replication	DNA replication origin binding
DAT6	GO:0006270	DNA replication	DNA replication initiation
DAT6	GO:0044786	DNA replication	Cell cycle DNA replication
DAT6	GO:0033260	DNA replication	Nuclear DNA replication

For functional annotation across multiple databases, see Supplementary Table S5.

Among the top 20 GO terms at DAT6, 11 were related to secondary metabolites, including flavonoids, phenylpropanoids, and glucosinolates. Additionally, four GO terms at DAT6 were associated with DNA replication (Table 1; Figure 3A).

KEGG pathway enrichment analysis revealed several pathways consistently enriched across all four time points, including “glyoxylate and dicarboxylate metabolism,” “carbon fixation in photosynthetic organisms,” and “alpha-linolenic acid metabolism.” In the meantime, some pathways were uniquely enriched at specific time points. At DAT0, the pathway “lectins” was exclusively enriched, while DAT1 showed specific enrichment in “carotenoid biosynthesis.” At DAT3, the “autophagy–other” pathway was uniquely enriched, and DAT6 exhibited significant enrichment in “DNA replication” (Figure 3B).

Photosynthesis-related pathways, including “photosynthesis proteins,” “photosynthesis - antenna proteins,” and “photosynthesis,” were notably enriched at both DAT0 and DAT3. Additionally, several antioxidant-related pathways were highlighted, such as “glutathione metabolism” at DAT1 and “flavonoid biosynthesis” at both DAT0 and DAT6 (Figure 3B).

### 3.6 Patterns of gene expression and functional enrichment analysis in heat-resilient cultivar AV-Ruby

**FIGURE 4:**
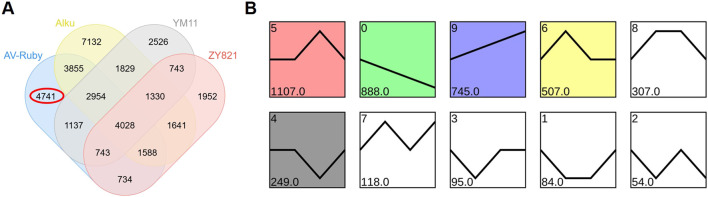
**(A)** Venn diagram of differentially expressed genes (DEGs) in heat-resilient *Brassica napus* cultivar (AV-Ruby) and heat-sensitive cultivars (Alku, YM11 and ZY821). 4,741 DEGs were assigned uniquely to AV-Ruby (red circle). **(B)** Expression patterns of AV-Ruby specific DEGs based on short time-series expression miner analysis. In each frame, the black line represents the overall expression trend of the grouped genes. The number in the upper left corner of each box indicates the profile ID, while the number at the bottom represents the number of genes assigned to that profile. The most populated expression profiles (profiles 5, 0, 9, and 6) were explored for distinct functional roles.

In this experiment, there were 4,741 DEGs specifically identified in AV-Ruby (Figure 4A) which were further classified by STEM analysis into ten profiles based on their expression patterns under heat stress. The most significantly enriched profiles (*p* < 0.05), accounting for over 10% of the grouped DEGs, were profile 5 (1,107 genes), profile 0 (888 genes), profile 9 (745 genes), and profile 6 (507 genes) (Figure 4B).

DEGs in profile 5 showed a late-response pattern, with no significant change at DAT0 but gradually increased from DAT1, peaked at DAT3, and then declined. In contrast, DEGs in profile 0 exhibited high expression level at DAT0, followed by a steady decline from DAT0 to DAT6, and DEGs in profile 6 exhibited an early-response pattern, with an initial spike in expression at DAT1, followed by a sharp downregulation. DEGs in profile 9 exhibited a delayed or gradual response, starting with low expression levels that increased over time.

To investigate the functional significance of gene expression changes, GO and KEGG enrichment analyses were performed on DEGs in profiles 5, 0, 9 and 6 (the most populated expression profiles).

**FIGURE 5:**
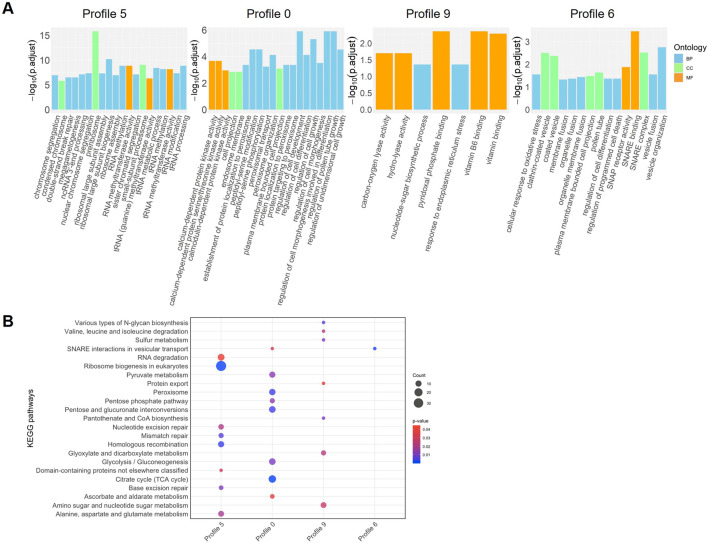
Functional enrichment analysis of the differentially expressed genes (DEGs) in heat-resilient *Brassica napus* cultivar (AV-Ruby) in profiles 5, 0, 9 and 6 of short time-series expression miner analysis. **(A)** Gene Ontology (GO) enrichment analysis. The GO terms were assigned to three categories: biological process (BP), cellular component (CC), and molecular function (MF). **(B)** Kyoto Encyclopedia of Genes and Genomes (KEGG) enrichment analysis. The size of the dot shows the number of genes in each KEGG pathway. The dot colour represents the *p*-value (red is higher, blue is lower).

In profile 5, 16 among the top 20 GO terms were related to RNA processing and DNA repair pathways, including “RNA methyltransferase activity” and “double-strand break repair via homologous recombination.” DEGs in profile 0, which were steadily downregulated over time, were predominantly enriched in cell growth and calcium-dependent kinase pathways, such as “regulation of pollen tube growth,” “regulation of cell development,” “calcium-dependent protein serine/threonine kinase activity” and “calcium-dependent protein kinase activity.” DEGs in profile 9, which demonstrated a gradual upregulation pattern, significantly enriched in seven GO terms, with the highest enrichment in “pyridoxal phosphate binding.” Meanwhile, DEGs in profile 6, which showed an early-response pattern with a sharp initial increase followed by a decline, were associated with vesicle trafficking, oxidative stress response, membrane fusion, and programmed cell death, suggesting their involvement in heat stress adaptation (Figure 5A).

KEGG pathway enrichment analysis further highlighted the functional differences among these profiles. DEGs in profile 5 were specifically enriched in pathways related to ribosome biogenesis and DNA repair, including “ribosome biogenesis in eukaryotes,” “mismatch repair,” “base excision repair,” “nucleotide excision repair,” and “RNA degradation.” DEGs in profile 0 were strongly associated with metabolic and antioxidant pathways, including “citrate cycle (TCA cycle),” “peroxisome,” and “ascorbate and aldarate metabolism“ Profile 9 was linked to various metabolic processes, such as “sulphur metabolism,” “glyoxylate and dicarboxylate metabolism,” and “amino sugar and nucleotide sugar metabolism.” DEGs in profile 6 were uniquely enriched in “SNARE (soluble *N*-ethyl maleimide sensitive factor attachment protein receptor) interactions in vesicular transport” (Figure 5B).

**FIGURE 6:**
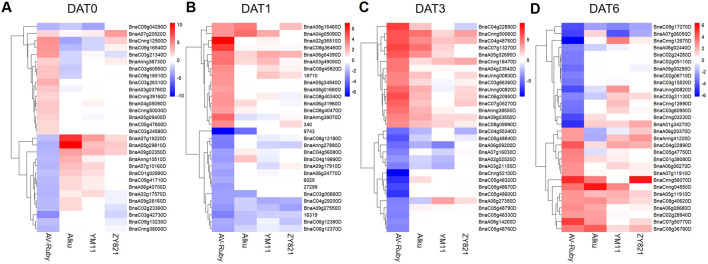
Heatmap based on clustering of expression values (|log_2_(fold change)|) of the top 15 up- and downregulated differentially expressed genes (DEGs) in *Brassica napus* cultivar AV-Ruby compared to changes in the same DEGs in heat-sensitive cultivars Alku, YM11 and ZY821 across four time points, **(A)** day zero of heat treatment (DAT0), **(B)** DAT1, **(C)** DAT3, **(D)** DAT6. Red indicates relatively increased expression, blue indicates decreased expression, and white indicates no change.

To further characterize the transcriptional responses underlying heat resilience, we examined the top 15 up- and downregulated AV-Ruby–specific DEGs across four time points (Figure 6; Supplementary Table S8). At DAT0, *BnaA03g03760D* (unknown protein) and *BnaA05g09400D* (light-harvesting chlorophyll-protein complex II subunit B1) (Supplementary Table S6) were markedly upregulated in AV-Ruby, with log_2_(fold change) values of 2.20 and 1.94, respectively, whereas both DEGs were downregulated in heat-sensitive cultivars Alku, YM11, and ZY821 under heat stress relative to the control treatment.

Several of the top 15 AV-Ruby–specific DEGs displayed significant expression changes across multiple time points. For example, *BnaA04g05090D*, a cytochrome P450-related gene (Supplementary Table S6), was upregulated at both DAT0 (log_2_[fold change] = 2.20) and DAT1 (log_2_[fold change] = 2.69). Similarly, *BnaC04g22890D*, associated with GENE SILENCING 3, showed strong heat-induced expression at DAT3 (log_2_[fold change] = 6.52) and DAT6 (log_2_[fold change] = 4.16). In contrast, *BnaC03g60950D*, related to *PsaB*, was significantly upregulated at DAT0 (log_2_[fold change] = 2.33) but notably downregulated at DAT6 (log_2_[fold change] = 5.06). Likewise, *BnaUnng00820D* (unknown protein, Supplementary Table S6) showed increased expression at DAT3 (log_2_[fold change] = −3.35) but downregulated at DAT6 (log_2_[fold change] = −4.23).

### 3.7 Quantitative real-time PCR (qRT-PCR) analysis

**FIGURE 7:**
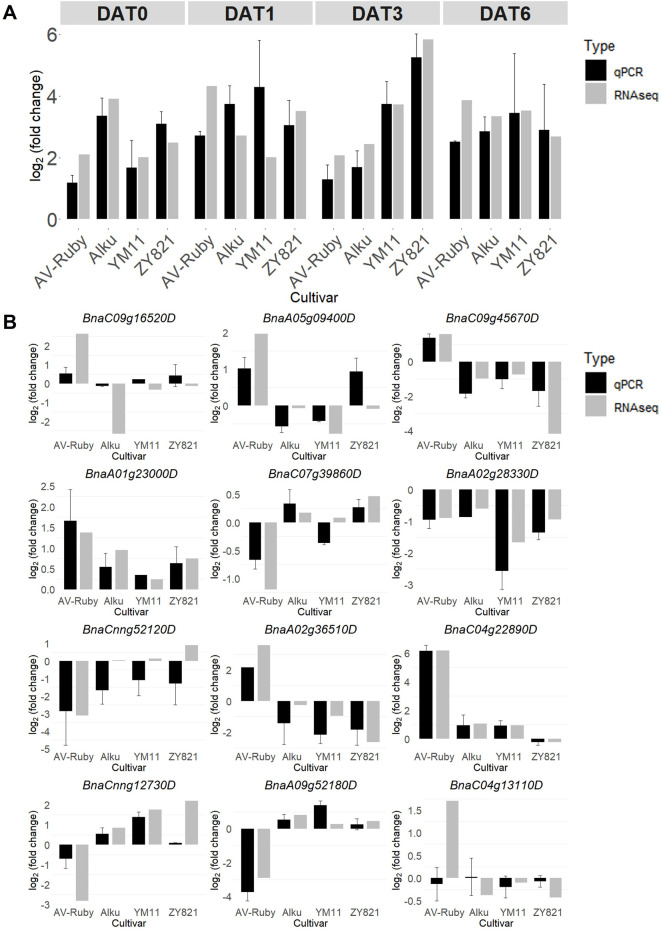
qRT-PCR validates the expression of **(A)** one common differentially expressed gene (DEG) (*BnaA10g20610D*) in four cultivars of *Brassica napus,* and **(B)** 12 DEGs randomly selected from four time points: day zero of heat treatment (DAT0), DAT1, DAT3, and DAT6 (for gene expression value and function information, see Supplementary Table S6). The transcript expression fold changes measured by qRT-PCR and RNA-seq are indicated by dark grey and light grey columns, respectively.

The expression patterns of thirteen DEGs randomly selected across four time points to validate the RNA-seq expression data were generally consistent with the RNA-seq results (Figure 7). As expected, the DEG that was common to all cultivars (*BnaA10g20610D*) showed consistent upregulation across all samples, confirming the RNA-seq findings (Figure 7A). Among the 12 randomly selected DEGs, seven (*BnaA01g23000D*, *BnaA02g28330D*, *BnaA02g36510D*, *BnaA09g52180D*, *BnaC04g22890D*, *BnaC09g45670D* and *BnaCnng12730D*) exhibited similar expression trends between qRT-PCR and RNA-seq. However, five genes (*BnaC09g16520D*, *BnaA05g09400D*, *BnaC07g39860D*, *BnaCnng52120D* and *BnaC04g13110D*) displayed discrepancies in expression direction. For example, *BnaC09g16520D* was downregulated under heat stress in the RNA-seq data in YM11 and ZY821 but upregulated in the qRT-PCR analysis (Figure 7B).

## 4 Discussion

Four cultivars of *B. napus* varied in their response to heat stress during the first 7 days of flower and pod development after pollination on the main stem. Cultivar AV-Ruby showed high heat-resilience, defined as the ability to maintain seed yield in pods developing at buds on the main stem inflorescence exposed to heat stress (Hu et al., 2025). That is, AV-Ruby suffered no significant reductions in seed yield in pods that developed from the four hand-pollinated flowers, both in this study and in a previous study (Hu et al., 2025). The other three cultivars—Alku, YM11 and ZY821—were more sensitive to heat stress as observed by a significant reduction in seed number in these pods (Figure 1). These results confirm that heat stress during the post-pollination stages severely reduces the number of seeds formed rather than seed size (Brunel-Muguet et al., 2015; Lohani et al., 2021; Wu et al., 2021; Mácová et al., 2022; Hu et al., 2025).

In this time-series transcriptomic experiment, we were careful to ensure that flowers were exposed to the same temperature regimes at the same age and position on the main stem. Only three HSP-related DEGs were consistently upregulated under heat stress in all four cultivars at all four time points. This supports the function of HSP genes as core components of a general heat stress response (Bourgine and Guihur, 2021). Two of these were sHSPs, which are known to mitigate heat-induced damage by reducing reactive oxygen species (ROS) levels and alleviate oxidative stress in plant cells (Sun et al., 2002; Wu et al., 2022). *BnaC04g12620D* encodes a 70-kDa HSPA8, a protein with limited documentation in plant heat responses, warranting further investigation. The importance of HSP-related genes was also shown in a previous proteomics analysis across the same time series, where three HSP-related DAPs (corresponding to *BnaC02g46730D*, *BnaC03g78270D*, *BnaA05g12530D*) were significantly upregulated in all cultivars and time points (Hu et al., 2025). These three HSP-related genes were also upregulated in this transcriptomic study under some but not all conditions (Supplementary Table S6).

The highest number of DEGs was observed at DAT3 (Figure 2A), whereas in a previous proteomics analysis across the same time series the highest number of DAPs was detected at DAT6 (Hu et al., 2025). This suggests that the transcriptomic response occurs earlier than the proteomic response. Post-transcriptional regulatory processes are likely to cause a temporal lag between gene expression and protein accumulation. Since samples for both proteomic and transcriptomic analyses were collected at the same time point, such differences in response between mRNA and protein levels are to be expected.

The increased number of DEGs at DAT3 may be associated with thermopriming, a phenomenon in which prior exposure to heat stress enhances subsequent stress responses (Bäurle, 2016). Similar findings have been reported in other transcriptomic studies, where heat pre-exposure triggered a broader activation of heat-responsive genes (Gao et al., 2021). In rice, heat-primed samples displayed nearly twice as many differentially expressed transcripts as unprimed samples (Kushawaha et al., 2021). These memory genes, which sustain activation after an initial stress event, are linked to acquired thermotolerance (Lämke and Bäurle, 2017). However, in Jedličková et al. (2023), where transcriptomic samples were collected at comparable stages to our experiment, the highest number of DEGs was observed at the ovule stage, while fewer DEGs were identified at the later eight-cell and globular stages. This difference may be because their samples were exposed to heat stress prior to flowering; thus, samples collected at different stages experienced the same duration of heat exposure, leading to a different expression pattern compared to our results.

Time-series transcriptomic studies have revealed an association between the drought stress response and the circadian clock in foxtail millet (Yi et al., 2022), and an early response of the MAPK signaling pathway in a heat-tolerant rice cultivar (Cai et al., 2023). This is the first time-series analysis of transcriptomic responses to heat stress in *B. napus* during the early reproductive stage. In this study, we analysed four time points during 1 week of heat treatment to characterise transcriptional dynamics and found that distinct heat-response pathways were activated at different times. A complex and dynamic regulatory network of expressed genes was associated with heat stress responses (Figure 3).

At DAT0, eight of the top 20 most enriched GO terms were related to cell growth or pollen tube growth (Figure 3), consistent with the findings of Poidevin et al. (2021), who reported similar enrichments during the heat response at the pollen tube growth stage in *Arabidopsis*. This result likely reflects the impact of heat stress on key reproductive developmental processes, such as pollen tube growth and double fertilization (Parry et al., 2025). At DAT1, pathways related to unfolded protein binding (heat-shock response) were significantly enriched, consistent with previous reports in *Arabidopsis* (Zhang et al., 2017), citrus (Balfagón et al., 2023), and *B. napus* anthers (Lohani et al., 2022). Jasmonic acid metabolic process was also enriched at this timepoint, aligning with findings in garlic, where jasmonic acid accumulation was observed following heat stress (Yang et al., 2025). By DAT3, pathways associated with photosynthetic electron transport and toxin metabolism were activated, suggesting mechanisms to mitigate oxidative stress. In addition, autophagy was significantly enriched only at DAT3. This pathway functions in protein degradation, targeting heat stress-related proteins such as aggregates and chaperones for removal during heat stress (Zhou et al., 2014; Zhang et al., 2017; Li et al., 2024). At DAT6, the dominant pathways included DNA replication, cell wall biogenesis, and secondary metabolite biosynthesis (e.g., glucosinolates), indicating a transition toward recovery and structural reinforcement.

Photosynthesis and light stress-related pathways were commonly enriched at DAT0, DAT1, and DAT3, suggesting an interplay between heat and light signalling pathways. Such interactions are well-documented in plants responding to dynamic environmental changes (Legris et al., 2017; Qi et al., 2022). A similar trend was observed in another *B. napus* transcriptomic study, where photosynthesis-related pathways were significantly enriched in heat-stressed developing seeds (Jedličková et al., 2023). It is possible that these pathways are also reacting to the infrared radiation that may be elevated in the heat treatment.

In AV-Ruby, the differential expression patterns across time points suggest a finely regulated heat stress response (Figure 4). The DEGs in profile 5 (late response pattern) of AV-Ruby were significantly enriched in homologous recombination repair pathways (Figure 5). Heat stress is known to induce DNA single-strand and double-strand breaks (Han et al., 2021). These breaks may accumulate to a threshold level, triggering DNA repair mechanisms (Han et al., 2021). This DNA damage repair response could be a critical defence mechanism contributing to AV-Ruby’s heat-resilience.

The early response pattern of AV-Ruby (profile 6) showed upregulation of genes related to oxidative stress response and vesicle trafficking (Figure 5), which is consistent with a study in *Arabidopsis*, where 6-h heat shock activated more oxidative phosphorylation genes than prolonged heat exposure (Wang et al., 2020). These findings highlight the complexity of heat stress adaptation. Additionally, genes in profile 6 were uniquely enriched in the soluble N-ethylmaleimide-sensitive factor attachment protein receptors (SNAREs) vesicular transport pathway, which related to vesicle trafficking (Gu et al., 2020). Vesicle trafficking has been linked to stress adaptation, with various SNAREs participating in abiotic stress signalling (Singh et al., 2016; Pan et al., 2019; Kwon et al., 2020).

The downregulation of calcium-dependent kinase pathways in profile 0 of AV-Ruby suggests a shift from early stress signalling to long-term acclimation (Figure 5). Calcium signalling is one of the earliest stress responses, with Ca^2+^ acting as a secondary messenger in stress perception and signal transduction (Kang et al., 2023). The observed suppression of these pathways at later time points indicates that initial stress signals may trigger a cascade leading to prolonged adaptation mechanisms. Additionally, AV-Ruby exhibited strong antioxidant defences, as evidenced by the enrichment of TCA cycle, peroxisome activity, and ascorbate metabolism pathways. Antioxidant defence pathways were also important in a proteomic analysis over the same time points (Hu et al., 2025) and is consistent with previous studies linking enhanced antioxidant activity to heat tolerance in plants (Sairam et al., 2000; Wang et al., 2014).

Genes in profile 9 of AV-Ruby were highly enriched in pathways associated with ER stress response (Figure 5), which plays a crucial role in maintaining protein homeostasis under heat stress (Singh et al., 2021). The upregulation of ER stress-related genes suggests that AV-Ruby employs molecular chaperones and the unfolded protein response to manage misfolded proteins and maintain protein stability under high temperatures. This mechanism improves cellular survival during heat stress, and was also reported in a parallel proteomics study (Hu et al., 2025).

Genes that exhibited distinct expression patterns between heat-resilient cultivar AV-Ruby and the heat-sensitive cultivars represent potential targets for future studies aimed at elucidating the molecular mechanisms of heat tolerance, and could serve as valuable molecular markers for screening and breeding heat-tolerant canola cultivars. For example, a cytochrome P450-related gene was upregulated at both DAT0 and DAT1 in AV-Ruby, whereas this was not observed in the three heat-sensitive cultivars (Supplementary Table S8). Cytochrome P450 enzymes have been reported to play important roles in plant stress responses by protecting cells from oxidative damage (Pandian et al., 2020). Transcriptomic studies in wheat (Qin et al., 2008), perennial ryegrass and tall fescue (Tao et al., 2017) have also shown strong induction of cytochrome P450 under heat stress. Its early and strong activation in AV-Ruby may contribute to its greater resilience to heat stress and warrants further investigation.

The identification of AV-Ruby–specific DEGs and their associated pathways offers valuable insights for breeding programs aiming to develop heat-tolerant *B. napus* cultivars. Key genes involved in oxidative stress response, ER stress regulation, metabolic reprogramming, and vesicle trafficking could serve as potential targets for genetic improvement. Future research should further investigate the underlying heat response mechanisms of these pathways and assess their potential applications in crop improvement.

## 5 Conclusion

This study provides a comprehensive time-series transcriptomic analysis in flowers at the second to fifth reproductive nodes on the main stem of four *B. napus* cultivars across 7 days of heat stress during the post-pollination stage. Cultivar AV-Ruby was heat resilient based on its ability to maintain seed set following heat stress exposure, whereas the other three cultivars were heat sensitive. Three HSP genes were consistently upregulated across all cultivars and time points, highlighting their fundamental role in heat stress response. Pathways related to DNA repair and antioxidant defence were enriched in AV-Ruby according to the GO enrichment, KEGG enrichment, and expression trend analyses. This suggests potential mechanisms contributing to heat stress resilience in AV-Ruby. These insights advance the molecular understanding of heat adaptation and identify priority targets for breeding heat-resilient canola cultivars. The dynamic expression profiles provide a foundational resource for optimizing heat stress response networks in *B. napus*.

## Data Availability

The datasets presented in this study are publicly available. This data can be found in NCBI with the accession number PRJNA1282636: https://dataview.ncbi.nlm.nih.gov/object/PRJNA1282636?reviewer=82jducrknms69qs422i6euuedl.
